# Pollen tube vs CHUKNORRIS: the action is pulsatile

**DOI:** 10.1093/jxb/erx207

**Published:** 2017-08-09

**Authors:** Simon Gilroy

**Affiliations:** Department of Botany, University of Wisconsin, Birge Hall, Madison, WI, USA

**Keywords:** Biological oscillations, calcium, CHUKNORRIS, kymograph, oscillation, pollen tube, pulsatile growth, tip growth, ultradian rhythms, wavelet analysis

## Abstract

This article comments on:

Damineli SC, Portes MT, Feijo JA. 2017. Oscillatory signatures underlie growth regimes in Arabidopsis pollen tubes: computational methods to estimate tip location, periodicity, and synchronization in growing cells. Journal of Experimental Botany 68, 3267–3281.

**The network of molecular components thought to regulate pollen tube growth presents an almost bewildering array of potential interactions.** Damineli *et al.* (2017) **have developed novel tools to help define the relationships between the kinetics of these proposed regulators and growth. They highlight both the insights and the challenges that extracting cause and effect from complex regulatory networks brings.**

Tip growth in plants represents the highly focused deposition of cell wall and membrane material in a small dome-like area at the tip of a growing cell. The wall behind this tip region becomes strengthened and does not allow growth (Box 1). The combination of a constant addition of new cell materials at the growing apex and a non-expanding subapical region then leads to the production of a hair-like cell extension such as a pollen tube or root hair. Sustaining this highly focused pattern of expansion requires intricate spatial and temporal regulation of the growth machinery. Over the past few decades a wide array of molecular components – including small GTPases, phospholipids (and their associated processing enzymes), the cytoskeleton, reactive oxygen species, and ions such as Ca^2+^ and H^+^ – have all been revealed to work in the cellular network that supports this process (reviewed in Fu, 2015; Gu *et al.*, 2003; Michard *et al.*, 2009; Gu and Nielsen, 2013; Sekereš *et al.*, 2015; Heilmann and Ischebeck, 2016; Michard *et al.*, 2017). The subcellular localization of these regulators fits well with the idea that each element should be driving growth restricted to the growing apex of the cell. For example, tip-focused gradients of Ca^2+^, phosphoinositides and small GTPases all correlate with periods of intense growth and dissipate as growth slows or stops.

**Box 1. Pollen tube growth**

Pollen tubes elongate by tip growth and represent some of the most rapidly growing cell types known. (A) The pollen tube tip expands while the shank is rigidified, generating a tube-like growth pattern. Scale bar=20 µm. (B) Expansion of the pollen tube tip and the activity of regulators thought to control this process can show oscillatory features, with phases of rapid expansion thought to trigger a feedback loop of Ca^2+^ influx and associated oscillations in activity of the growth machinery. The fine-scale mapping of growth and cellular activities in both time and space by Damineli **et al.** (2017) now challenges this model, requiring its extension to uncouple oscillations in the activity of cellular regulators of the tip growth machinery from these bursts of cell expansion.

**Figure:**
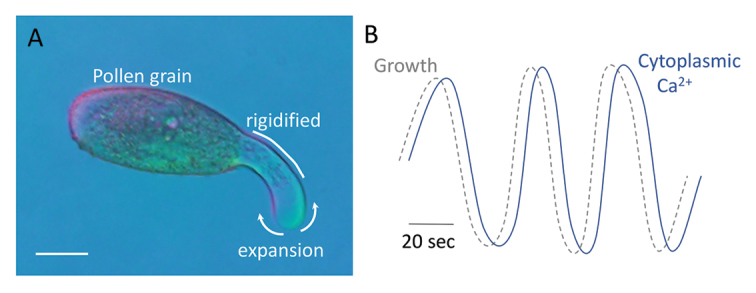

## Pulsatile growth and oscillating regulators

In addition to its spatial component, tip growth has a prominent temporal element showing an oscillatory character with periods of rapid elongation interspersed with periods of slower or arrested growth (Box 1). Such pulsatile growth is seen in both tip-growing pollen tubes (e.g. Feijó *et al.*, 2001; Holdaway-Clarke *et al.*, 2003) and root hairs (e.g. Monshausen *et al.*, 2007), suggesting that oscillatory phenomena may be a fundamental aspect of this type of cell elongation. Thus, models of the cellular networks driving the tip growth process must incorporate not only the identity of putative regulatory components but also their spatial and temporal characteristics. Correlating the dynamics of possible regulators of the tip growth machinery with both the spatial and temporal dynamics of growth has therefore been a logical focus of research towards placing each element within the larger network of cellular interactions that ultimately drive tip growth. For example, measurements of the Ca^2+^ gradient focused on the growing tip of pollen tubes and root hairs suggest that its peak occurs at the very tip of the expanding apex and is seen a few seconds after a pulse of growth. These observations have led to a model where the rapid phase of tip expansion leads to mechanical stress within the plasma membrane that opens stretch-activated, Ca^2+^-permeable channels. The associated Ca^2+^ influx drives a localized increase in cytosolic Ca^2+^ that in turn regulates a suite of Ca^2+^-dependent processes in this region to localize the growth process to the tip (Michard *et al.*, 2017). That is, the pulsatile nature of growth is inextricably linked to the cellular machinery supporting expansion.

Defining such spatial and temporal relationships with any kind of statistical rigor is a major challenge for researchers in the tip growth field. Therefore, Damineli *et al.*, (2017) have developed a novel, integrated analytical suite to help address these practical questions as to measuring correlation and assessing synchronization. These researchers have used image analysis of pollen tubes to extract data on tip growth dynamics, using a clever linear regression approach to increase their spatial resolution beyond the absolute X-Y limits of the raw data in their images (i.e. to ‘sub-pixel’ levels). These high-resolution measurements are then combined with a suite of algorithms based on wavelet analysis to provide correlations with periodicity and phase of other oscillatory features in the growing cell such as ion fluxes or the dynamics of fluorescent reporters. The combined package, the *C*omputational *H*euristics for *U*nderstanding *K*ymographs and A*n*alysis of *O*scillations *R*elying upon *R*egression and *I*mproved *S*tatistics (CHUKNORRIS), offers a new, quantitative and statistically rigorous way to approach these links between timing and position of changes. Importantly, this analytical approach provides a measure of the synchronicity (delay, phase relationship, joint periodicity) between multiple phenomena such as growth and ion flux even as these features vary with time.

## Models and mysteries

Such oscillatory phenomena are seen in pollen tubes grown *in vitro* in species as varied as tobacco, petunia and lily (e.g. Feijó *et al.*, 2001; Holdaway-Clarke *et al.*, 2003). However, Damineli *et al.* (2017) have used CHUKNORRIS to reveal that in Arabidopsis pollen tubes growing *in vitro*, oscillations are associated with slow or stopped growth. Steadily growing tubes showed little evidence of large amplitude oscillations in, for example, Ca^2+^ fluxes, instead possessing a stably increased basal level of this ion. As noted above, pulsatile behavior has been strongly linked to tip growth in many cell types and the phase relationships between such phenomena have been taken to reflect regulatory behavior and so used to drive models of the underlying molecular network. This current work coupled with observations of non-oscillating pollen tube Ca^2+^ dynamics *in planta* (Iwano *et al.*, 2009) raises the question as to the fundamental dynamics of the regulatory systems governing tip growth. Damineli *et al.* (2017) do demonstrate that specific frequency signatures are linked to specific growth regimes, just not the signatures predicted by many current regulatory models. Their work also raises the question as to what these tip-growth oscillatory phenomena reflect if not the dynamics of the regulatory network that is driving growth.

The answer is undoubtedly that biological processes are complex. The more we dissect them to their molecular underpinnings, the more we see that the control systems underlying most of biology reflect intricate networks of regulators and molecular machinery. These cellular components interact to provide both regulation and integration between processes through complex feed-forward and feed-back loops. Some of the dynamics of these molecular interactions reflect elegant control systems that monitor and adjust the biological process; others are simply a consequence of the kinetics that emerge from the architecture of the interactions within the complexities of the machinery. Just as the architecture of a guitar impacts the tone of an oscillating guitar string yet it is the musician who regulates this oscillation to produce a melody, so are the inherent dynamics of biochemistry tuned by cellular networks to produce the ‘music’ of growth.

Why does biology rely upon such complicated regulatory structures? In the terminology of modeling, these control systems reflect scale-free networks, much like the internet. A key feature of such systems is robustness: disruption of one component does not knock out the entire regulatory landscape (Albert *et al.*, 2000; Barabasi and Oltvai, 2004). With these robust network features come the many feed-back and -forward loops and interrelationships that lead to non-linear kinetics. How then to approach the daunting task of placing the multitude of cellular regulators already linked to tip growth into their respective network positions amid this sea of complex interactions? CHUKNORRIS provides one piece of the puzzle with a quantitative view of the dynamics and outputs of these regulatory elements. However, as Damineli *et al.* (2017) note, correlation does not alone identify causation. The challenge now is to integrate these kinds of measurements with molecular and biochemical analyses to provide a view of potential system topology. Combining these insights with biophysical measurements and models of the interactions between tube geometry and the cell wall (e.g. Kroeger *et al.*, 2011; Sanati Nezhad *et al.*, 2013; Liu and Hussey, 2014; Bidhendi and Geitmann, 2016; Hu *et al.*, 2016) should help place these molecular events into the physical features of a tip-growing cell, offering great promise in helping define the system as a whole that drives tip growth. This is a cutting-edge problem of quantification and modeling and CHUKNORRIS provides an important new tool for helping make this synthesis happen.
